# Unmanned Aerial Vehicles (UAVs) for Physical Progress Monitoring of Construction

**DOI:** 10.3390/s21124227

**Published:** 2021-06-20

**Authors:** Nicolás Jacob-Loyola, Felipe Muñoz-La Rivera, Rodrigo F. Herrera, Edison Atencio

**Affiliations:** 1 School of Civil Engineering, Pontificia Universidad Católica de Valparaíso, Av. Brasil 2147, 2340000 Valparaíso, Chile; nicolas.jacob.l@mail.pucv.cl (N.J.-L.); rodrigo.herrera@pucv.cl (R.F.H.); edison.atencio@pucv.cl (E.A.); 2 School of Civil Engineering, Universitat Politecnica de Catalunya, 08034 Barcelona, Spain; 3 International Center for Numerical Methods in Engineering (CIMNE), 08034 Barcelona, Spain

**Keywords:** construction site monitoring, physical progress, unmanned aerial vehicles, photogrammetry, BIM 4D

## Abstract

The physical progress of a construction project is monitored by an inspector responsible for verifying and backing up progress information, usually through site photography. Progress monitoring has improved, thanks to advances in image acquisition, computer vision, and the development of unmanned aerial vehicles (UAVs). However, no comprehensive and simple methodology exists to guide practitioners and facilitate the use of these methods. This research provides recommendations for the periodic recording of the physical progress of a construction site through the manual operation of UAVs and the use of point clouds obtained under photogrammetric techniques. The programmed progress is then compared with the actual progress made in a 4D BIM environment. This methodology was applied in the construction of a reinforced concrete residential building. The results showed the methodology is effective for UAV operation in the work site and the use of the photogrammetric visual records for the monitoring of the physical progress and the communication of the work performed to the project stakeholders.

## 1. Introduction

The construction industry is vital to the economy and represents a significant part of the gross domestic product of each country, regardless of its level of development [1]. Still, this industry historically has had low productivity rates and, even in the current era of digitalization, is resistant to emerging technologies, mainly owing to its traditional culture and high resistance to change; however, this situation is shifting [2].

The planning phase of a construction project is a critical stage, during which tasks are discretized, deadlines are established, and a master plan for project execution is created. This phase has been widely addressed by academia and industry, incorporating methodologies such as lean construction and tools such as the Last Planner^®^ System to establish orderly workflows at the beginning of the project, which must be monitored during the execution phase through different indicators [3]. Despite these tools, one problem remains at the construction site: how to measure and record the physical progress of the construction project. While managing progress and calculating the associated indicators are administrative tasks, the collection of information on the physical progress of construction from the job site is typically a manual, strenuous undertaking, supported mainly by subjective photographic material, which tends to accumulate as waste data within the site’s assets [4].

The preparation of a conventional progress report should be backed up with a photographic record, which is a set of views restricted to the photographer’s movement through a workspace full of obstructions and limited by the height and range of view from the camera [5]. In this sense, the development of unmanned aerial vehicles (UAVs) equipped with cameras has been well received in the construction industry, proving successful in the inspection of structures and workspaces and allowing site professionals to improve their visualizations of the sites [6,7].

Because the construction phase makes up a significant portion of the total project cost, project team members need to be apprised of the progress of the work. Therefore, a system is needed to provide task information (who is doing what task and in which location within the site), maintain optimal control of the workflow, and remotely visualize work progress and the evolution of the project’s construction status [8]. New methodologies oriented to construction virtualization, such as building information modeling (BIM), have improved the digital visualization of the construction site. However, these alone are not capable of capturing and displaying the project’s on-site work performance and construction progress, so manual and periodic reports and photographic records are still required for a digital time reconstruction [9].

The generation of a three-dimensional (3D) support of the construction site is possible by means of laser scanning technologies, which deliver high-precision and -accuracy models at high costs. On the other hand, computational advances in photo processing allow for increasingly efficient and user-friendly algorithms and software capable of generating 3D models from two-dimensional (2D) images obtained by conventional cameras. Thus, photogrammetry is presented as a less expensive and simpler alternative to its counterpart, the laser scanner, through the use of images captured with UAVs [10].

Different workflows have been proposed in the literature for the processing of site images by photogrammetry to help monitor construction progress. However, no methodology covers the complete flow from the UAV operation on-site to the data processing for the integration as a monitoring tool. Additionally, current approaches do not establish guidelines for the flight and periodic recording of a structure under construction.

The objective of this article was to formulate a holistic methodology for monitoring the physical progress of the construction site, from a strategy for the repetitive structure inspection using an UAV to the generation of a virtual replica of the current state of the construction site and its comparison with the four-dimensional (4D) BIM model. The technique will improve the follow-up and visualization of the work performed and detect gaps with the schedule of the project.

### 1.1. Research Methodology

**Figure 1:**
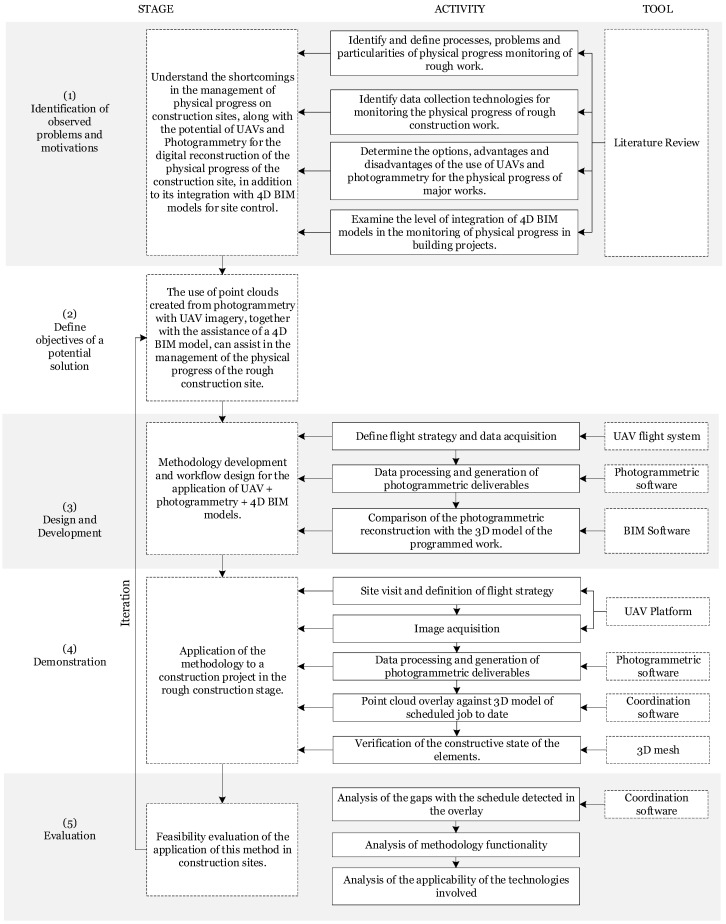
Research methodology.

For the development of this research, the design science research method (DSRM) was used as a basis, which shows the whole process of justification, development, and testing [11]. Tool and activity components were included to explain the research conducted in an expanded form. The research methodology was organized in five stages: (1) identification of observed problems and motivations, (2) definition of the objectives for a potential solution, (3) design and development, (4) demonstration, and (5) evaluation. Figure 1 shows the research activities and tools that describe the scenario for each stage.

In the first stage, a literature review was conducted with the following goals:

- identify and define processes, problems, and particularities of physical progress monitoring of major works;
- identify technologies for data collection from the construction site;
- determine on the options available with UAVs and photogrammetry for the digital reconstruction of real scenarios; and
- examine the level of integration of 4D BIM models for construction site monitoring.

Scientific articles and conference proceedings based on Web of Science and Scopus search engines were collected from the last 20 years, along with technical reports, books, and manuals on the indicated topics using the following keywords: construction site monitoring, physical progress monitoring, UAVs, photogrammetry, and BIM 4D. Ninety-one articles were found, among which high-impact journals were prioritized. The study sought out those articles that focused on UAVs in the construction industry, short-range photogrammetry, and the integration of point clouds in 4D BIM models, resulting in 54 references.

In the second stage, the objective of a potential solution was defined based on the literature review: to use reality models created from photogrammetry with UAV imagery, together with the assistance of a 4D BIM model, to help manage the physical progress of a construction site.

In the third stage, a workflow for monitoring the physical progress of construction sites was designed. By studying remotely piloted flight systems, guidelines could be drafted for UAV operation in the construction environment aimed at obtaining a photographic record of the worksite. Based on the work with photogrammetric software, a flow was developed to process the data acquired. With the help of coordination software, instructions were then generated to integrate the photogrammetric deliverables in the 4D BIM environment for the comparison between the actual and scheduled work progress.

In the fourth stage, the designed flow was applied to a case study in a construction project in the rough construction stage. This typical case corresponded to a high-rise residential building, in which the construction progress could be seen in the vertical growth of the regularly shaped structure, facilitating the design of a flight routine for the periodic inspection of the project. The designed methodology is expected to be applied to a more complex structure by adapting the aircraft operation guidelines according to its shape and location. Finally, in the fifth stage, the methodology was evaluated by assessing its applicability and functionality in the internal monitoring of a construction project.

### 1.2. Literature Review

#### 1.2.1. Traditional Construction Monitoring

Monitoring during construction includes real-time observations of all process dynamics, such as cost, time, materials, and physical progress, throughout the life of a construction project. By monitoring progress, deviations from the original plan can be detected, and corrective measures and other actions can be taken in a timely manner to keep the project on schedule [12]. Construction projects are costly; each variable mentioned above has an associated cost per unit of time, and each project will be subject to an exact time period determined by contract and a penalty associated with the deviation from this period, increasing project costs [13]. Therefore, construction professionals are motivated to seek the optimum performance for each work package, encouraging good management practices to maintain adequate control of the work [14].

In the construction industry, the traditional approaches of monitoring construction work are difficult to change [15]. The wide variety of projects with different tasks and processes results in a diversity of problems associated with the construction process [16]. In the face of a culture resistant to change and lacking long-term thinking, professionals are reluctant to opt for innovative solutions.

Traditionally, the monitoring of construction work has incorporated simple tracking elements, such as the use of a logbook, in which major events and daily progress are recorded on-site. This document can be complemented with tools, such as control sheets, measurement diaries, progress charts, and site-meeting minutes, to help monitor construction progress, update the construction schedule, and, thus, minimize delays in project deliveries [17].

To visually support the construction progress, photographs and video obtained by on-site team members have been used extensively to complement the logbook; the accessibility to cameras has exponentially increased the daily number of images that can be obtained of a work area [18] Therefore, several mobile applications have emerged allowing the creation of digital logbooks that facilitate the management and display of information from the captured images.

Site photographs are used for both progress monitoring and the visualization of construction operations, productivity measurements, accident investigation, troubleshooting, and quality control [5]. This practice is becoming a valuable resource for most of the operations related to the project. However, photographs have significant limitations associated with the camera’s image sensor, whose aperture and maximum resolution define a direct relationship between the proximity to the lens and the detail of the resultant image. Additionally, the range of view, or the amount of space that can be captured in the image, increases with the camera’s distance to the captured elements. A unified and detailed view of the general panorama of the work is, therefore, difficult to attain, and more photographs are taken from different angles of the space of interest to compensate for these limitations [19]. The photographer may choose to generate an exhaustive record of the worksite, moving around the sector taking numerous photographs to capture all the necessary details. The result of this decision is a large set of image files whose quality may be diminished by occlusions in the field [20], the restriction of the camera’s range of view, and the limited movement of the photographer around the worksite. A significant amount of potentially disposable information can result, which must be subsequently reviewed by the technical office professionals, making progress monitoring an extensive and exhausting task [4].

#### 1.2.2. New Methodologies and Tools for Monitoring Construction Projects

New methodologies and tools have been incorporated into the construction industry to better the monitoring and control of projects, such that management processes can improve and timely actions can be taken, with a focus on correcting any identified problems [21]. Lean construction is a philosophy of lossless production and management practices applied to construction, which provides a series of tools to optimize processes. In this sense, the Last Planner System proposes weekly meetings to analyze the work completed and to plan short-, medium-, and long-term tasks, based on what “must” be done, “can” be done, and those that finally “will” be done [3]. These meetings, in which representatives from all project specialties participate, should include daily reports and visual information recorded on-site, allowing for feedback on the work done and those tasks that were performed or completed, which aids in future planning [22].

Another approach is BIM, which is a collaborative work methodology that promotes the integration of processes and professionals from the different specialties involved in a project, interconnected around a parametric 3D model that allows the visualization, coordination, and analysis of construction projects [23,24]. This 3D model is a deliverable of the BIM methodology and contains all the geometric information related to the project throughout its life cycle. A monitoring plan can easily be established for the construction process since the model provides a complete 3D visual representation of the project plan [14].

Incorporating the fourth dimension of time, a 4D model integrates the scheduling of a project’s construction phase, including the date each element of the BIM model must be built. The coordination between the breakdown structure of the work schedule and that of each of the elements of the 3D model is required [19,25]. The management of a construction project using a 4D BIM model is an improvement on the traditional way of monitoring the work performed. The BIM model is a helpful planning and control tool because one can easily determine who executes a specific work package, along with the specific location and date [26]. The projected information is compared with the work progress, so the site information has to be surveyed and then digitized in the BIM model or other management tool. The 4D BIM management improves project control but not information retrieval from the site with the massive number of images and videos that soon become obsolete [22].

#### 1.2.3. Current Deficiencies and Challenges in Construction Site Monitoring

Methodologies and tools for project management and control can increase the efficiency of monitoring processes but do not successfully address the challenges with information collection from the construction site, data processing, subsequent presentation to project participants, and schedule upkeep [26].

In these processes, the visual support has become a relevant input to complement the construction progress reports but is conditioned and limited to how the images are obtained. Variables such as camera quality and characteristics and movement limitations of the photographer affect the efficiency of the image capture [27].

Thus, photographing becomes a subjective task, subject to a series of decisions at the discretion of the photographer, who needs to perform the following tasks: (1) select relevant on-site scenes, (2) choose a distance to the target, sacrificing resolution for the range of view and facing possible obstructions, and (3) capture a sufficient number of views to fully deliver the information [5]. The photographer typically generates an exhaustive and selective record of photographs with valuable information to deliver to all project stakeholders and serve as a backup for the progress log. However, because of the aforementioned problems, the record is in the form of an excess of files that must undergo a second review and selection process by the technical office. Thus, both the record generation and its review consume valuable work time of the professionals on-site and in the office. Despite this effort, the result is a deficient, subjective, and inaccurate representation of visual information on the current status of the work performed on-site. The stored content cannot be easily accessed for progress reports and planning meetings, potentially becoming disposable information that hinders decision-making processes [9].

#### 1.2.4. Image Capture and Processing Technologies under Construction

To improve the visualization of the work carried out on construction sites, fixed cameras have been placed on-site to monitor progress from strategic static and dynamic points, such as the tip of a crane [28,29]. However, the integration of UAVs has significantly advanced as a vital contributor to the construction process monitoring chain [30]. UAVs started off as military tools but have become devices for professional use, presenting diverse applications in civil engineering [6,29]. Microchip technology, GNSS receivers (Global Navigation Satellite System), and inertial measurement units (IMUs) have allowed the development and proliferation of these devices, which are capable of carrying a camera and traveling to a specific point in space. A wide range of remotely piloted aerial vehicles with integrated cameras can transmit visual data in real time to the pilot [31].

The use of UAVs is a game changer in the inspection of the state of the construction site since the capacity of aerial displacement allows multiple points of view of a given target together with its surroundings. UAVs equipped with at least four rotors have the freedom of 3D displacement, which eliminates the view limitations typical of traditional on-site monitoring and allows, in many cases, circumventing terrain occlusions. The range of view of the acquired images is widened, and the height is no longer limited [10]. However, limitations of UAVs in construction projects are associated with image resolution and communication stability, deficiencies that are becoming less relevant as technology advances [7].

Construction projects, due to their size and complexity, add up to numerous obstacles when it is necessary to carry out a data collection campaign [32]. Along with this, the difficulty of developing a proper flight planning to capture the data of the structure being studied arises. There are restrictions specific to the environment where the UAV is operating that affect the stability and operation of the drone, as well as the characteristics of the structure that is required to model [33]. In photogrammetry applications, accuracy is a central theme and depends on the suitability of the captured data, in this case, the photographs. Thus, in order to obtain the desired accuracy, the complexity of the object of study must be taken into account, especially the vertical elements that are the most difficult to represent. Properly characterizing these elements will require planning the capture of photographs at different angles [34].

With the photographs captured from the UAVs, a digital 3D model can be constructed using photogrammetry techniques [35], using a sequence of computational algorithms that detect points on the captured surfaces to locate in a virtual environment, generating a point cloud of the reconstructed scene [36].

Initially, photogrammetry was developed with images acquired from terrestrial cameras, which were subject to all the abovementioned deficiencies. Photographic cameras were eventually incorporated into different aerial devices, finally achieving stable reconstructions from images acquired from airplanes, hot air balloons, and, later, satellites. Despite the low-resolution images, the satellites could detect the entire electromagnetic spectrum and help generate digital elevation models (DSMs) for various uses [37]. Photogrammetry has become such a powerful tool that 3D environments can be reconstructed from unconnected tourist photographs found on the internet [38]. Together with georeferencing systems, photogrammetry can obtain visual information from large distances with millimetric precision for engineering projects. 

The processing of the photographs is carried out by software that applies a series of algorithms to identify and position 3D points captured in the set of photographs [39], thus constructing the point cloud. Most 3D reconstruction software uses the SIFT algorithm (scale-invariant feature transform algorithm) followed by the SfM algorithm (structure-from-motion algorithm) and, finally, the MVS algorithm (multi-view stereo algorithm) [40].

The SIFT algorithm is applied indistinctly to each image and allows the extraction of features from the photographs by locating areas where the quality of the processed image is key to obtain a denser point distribution; this is the starting point for the photogrammetric reconstruction. The SfM algorithm triangulates the points obtained by SIFT, using control points, i.e., surface points displayed in the set of photographs of known coordinates and manually entered in the processing, or using the coordinates of position and rotation (pose) offered by the IMU and GPS sensors of the device in each photograph [41]. While traditional photogrammetry requires a rigid registration of the camera pose or the position of the control points, SfM estimates these values through the identification and interpretation of the same characteristic points obtained by SIFT in the image set, with the requirement that a key point is identified in a minimum of three different images [42]. Only the pose reported by the UAV is used to iterate and refine the referencing of all points in the scene [10]. The result of this algorithm is a low-quality, sparse point cloud, which is densified and enhanced by MVS [43], significantly increasing the number of points in the cloud, generating a deliverable with metric and visual information of the recorded surface [36]. A variety of software exists on the market today capable of applying the aforementioned algorithms sequentially and automatically with user-friendly interfaces [44].

Photogrammetry can be applied to any set of images of a given area, regardless of its scale, and, therefore, has been described as short-range, which allows the reconstruction of surfaces of less than 100 m^2^ and, thanks to advances in camera calibration, presents better precision for industrial inspections [45].

Data acquisition using UAVs for the reconstruction of a 3D scene is subject to parameters associated with the characteristics of the images obtained and the operation of the device. This has been extensively studied by academia. Hoppe [46], for example, defined three factors for a sufficient configuration of the set of photographs: (1) an upper limit for the uncertainty of the points to be reconstructed, (2) a minimum of overlap between a pair of consecutive images, and (3) the totality of the parts of a surface to be captured covered by the photographs.

The uncertainty of points to be reconstructed (1) will be conditioned by the resolution of the captured digital image, as this is a scaled view of the real captured surface. The real size of each pixel of the image projected on the surface can be determined. The uncertainty factor, called ground sample distance (GSD), which is calculated according to Equation (1), defines the minimum differentiable dimension of the surface in the image by relating the digital size of the image to the real surface captured through the camera parameters of sensor size, focal length, and the distance to the surface.

(1) $$GSD\left[\frac{m}{\mathrm{pixel}}\right]=\frac{\;Sensor\;size\;\left[\mathrm{mm}\right]}{Focal\;length\;\left[\mathrm{mm}\right]}*\frac{Tarjet\;distance\;\left[m\right]}{Image\;size\;\left[\mathrm{pixel}\right]}$$

This parameter of the digital image does not represent a reliable measure of the 3D reconstruction made from the images taken. However, the algorithms used in the reconstruction rely on the number of characteristic points perceived in each image; so, a lower GSD indicates a higher probability of finding characteristic points in that image [47].

Images obtained from the surface should have adequate overlap between adjacent views (2), which will allow more points to be identified by the reconstruction algorithms [48]. Although there is no optimal overlapping of images, several authors have considered a minimum of 30% for urban mapping [49], 65% for digital terrain models [50], and 75% for short-range reconstructions [51]. Point (3) implies only surfaces captured by the images can be reconstructed, requiring clean views without occlusions or shadows [48]. The UAV needs to fly over the captured space at the specified time and withstand the climatic factors of humidity and wind speed that may affect or impede its operation.

## 2. Methods

**Figure 2:**
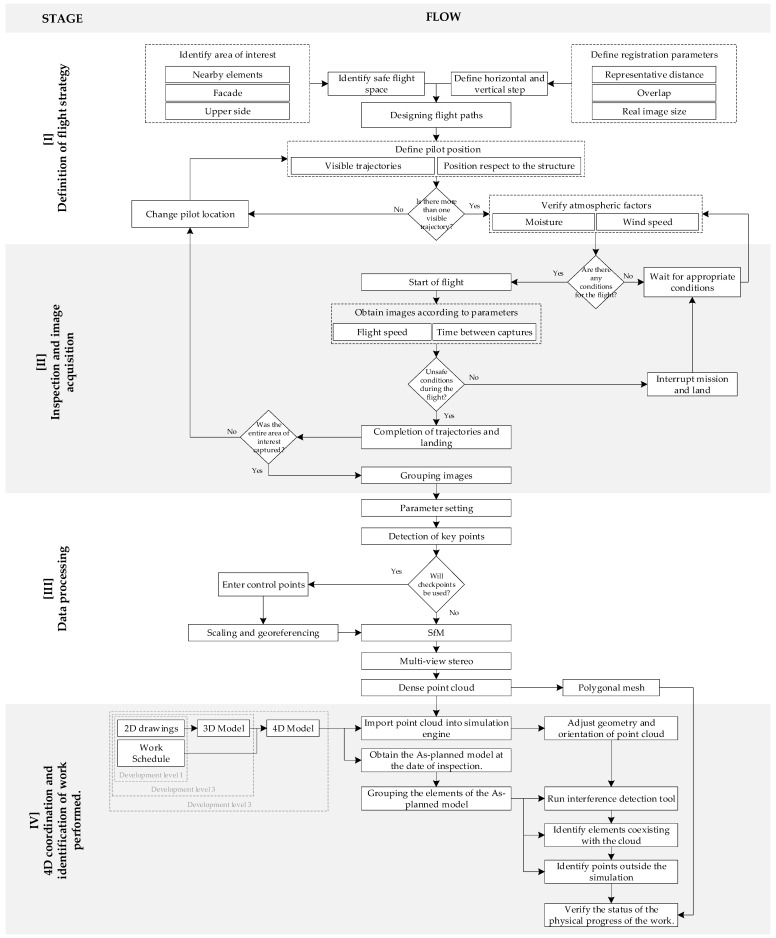
Flow chart of the proposed work progress monitoring inspection methodology.

Despite the existing methodologies for monitoring construction progress using photogrammetric models, a comprehensive flow cannot be created to fully encompass the integration of a UAV as a monitoring tool. Several case studies have explored the advantages of photogrammetric models applied to construction monitoring; however, the studies focused on only a part of the process involved in the integration of these technologies for construction project communication. Therefore, to monitor on-site work progress using a 4D BIM model and UAV imaging, the workflow, as shown in Figure 2, was proposed, which was composed of four stages: (1) definition of a flight strategy with fixed trajectories for the UAV path in each inspection, (2) definition of the parameters that help guide the pilot to perform the inspection and acquisition of images of the surfaces of interest, (3) processing of the data collected during the inspection to generate a photogrammetric model with visual information of the state of the work performed, and (4) model incorporation into a coordination software for comparison with the state of work scheduled for the day of the inspection.

### 2.1. Definition of Flight Strategy

To improve the visualization of the work carried out on construction sites, cameras on UAVs are used to perform repetitive inspections of a building project. Predefined trajectories have to be generated to obtain sequential images of the workspace at any stage of the project. For the pilot to easily traverse without the need for advanced flight skills, the trajectories need to be either horizontal or vertical. The 2D trajectory allows the pilot to maintain control of the aircraft’s travel, with only planar movement, and take manual photographs through the shutter without having to stop the movement of the device.

Because of the uncertain topography around construction sites, the location of the surface where the scene of interest is to be reconstructed is required. The project location needs to first be identified, along with surrounding elements, natural or artificial, that limit the flight space, allowing for a safe flight space. Proximity is maintained to achieve an appropriate but safe resolution to avoid accidents. The lengths of dynamic elements such as cranes can help to define a lower elevation limit for the flight to avoid collisions; elevations of 15–20 m are traditionally used for inspections of structures by UAVs [48].

**Figure 3:**
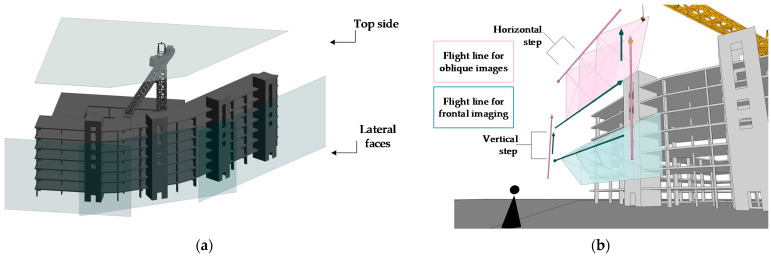
(**a**) Definition of the top face and lateral faces according to the shape of the structure. (**b**) Flight paths in front of each face, horizontal and vertical steps.

To spatially order the scene to be reconstructed, the “top face” is defined as the view of the pilot flying over the workspace above its tallest element and facade as the side views of the structure. Figure 3a shows the lateral faces at the front of the facade of a structure under construction and the upper face over the workspace.

In front of each identified face, a flat trajectory needs to be defined to capture the entire surface through sequential and overlapping photographs [48]. The aircraft must focus on the face of the surface to be captured and then move horizontally in a direction parallel to the face, making a sweep from end to end, then moving to the next elevation or flight line until the entire surface is captured. Additionally, the trajectory must include flight lines allowing for oblique photographs of the surfaces to visualize two adjacent faces in a single image so that the algorithm can identify and triangulate the key points identified on each surface. The top face contains more visual information about the work performed and more elements belonging to the activities of the schedule. To obtain information about the depth of the internal elements of this face, the frontal trajectory should include flight lines of oblique photographs of the internal surfaces of this face, as shown in Figure 3b. The quality of the photogrammetric reconstruction of these elements will depend on the quality of photographs obtained at different points of view [47,51,52].

The facade of the structure, according to its shape, may contain a number of lateral faces (Figure 3), which should be chosen such that, when tracing a flat trajectory in front of each face, the distance from the UAV to the surface of interest does not vary significantly. The photographic record should contain overlapping images to identify and triangulate the points of interest. Since the images are taken from a 2D trajectory traversed by the UAV, the actual distance between the image sensor and the focused surface varies. Therefore, a representative distance between the UAV trajectory and the surface of interest must be defined based on the safe flight space, as identified above, and the upper-resolution limit of the photographs obtained by the GSD [46]. This distance, together with the camera parameters, allows us to calculate the real size of the image obtained by the device and projected onto the captured surface using Equation (2). With the rectangular shape of most sensors, the projection retains this shape and has both a horizontal and vertical real size.

(2) $$Real\;size\left(h,v\right)\left[m\right]=Representative\;distance\;\left[m\right]*\;\frac{Sensor\;size\;\left(h,v\right)\left[\mathrm{mm}\right]}{Focal\;length\;\left[\mathrm{mm}\right]}$$

For short-range photogrammetric reconstructions, different values can be used for vertical and horizontal overlaps, the latter being typically greater, as this is the direction traveled for sequential imaging [51]. The desired overlap *T* in the sequence defines the distance, or step, of the UAV between one capture position and the next in the flight line. Since horizontal and vertical overlaps can differ, horizontal and vertical steps are defined according to Equation (3).

(3) $$Step\;between\;captures\;\left(T\%\right)=\left(1-\frac{T}{100}\right)*Real\;size\;\left(h,v\right)\left[m\right]$$

The design of the flight strategy considers at least one trajectory to be followed by the UAV for the top face and facade with frontal photographs. Oblique photographs are required where the adjacent faces are present. The lateral faces are then supported with at least one vertical line of oblique photographs for the edge between the adjacent faces, focusing on this plane of encounter to obtain perspective views of both consecutive faces.

The trajectory on the complex top face has to be thoroughly complemented with oblique photographs. The pilot must choose an angle that grasps the depth of the constructive elements of this face and generate horizontal flight lines using the same horizontal step defined for the nadir photographs.

Based on the proximity to the structure and the manual nature of the image capture, the pilot’s position for the inspection requires the UAV to be in view during the execution of the flight trajectories. Therefore, the location of the pilot requires the visibility of at least two lateral faces of the work. The pilot can then travel two visualized trajectories from a single position in a single cycle of takeoff, flight, and landing, as allowed by the battery life. A position(s) should also be chosen that allows visibility of the trajectory over the top face, a condition that may mean that the pilot has to complete the upper trajectory from two different positions. 

The design of the flight trajectories is useful to generate photographic records expeditiously and repetitively. However, the flights are subject to the climatic conditions of the project site. Humid climates, where there is a high probability of rain, and/or windy sectors are risky for a short-range flight, and the time and day of the inspection must be carefully programmed to minimize the possibility of rain, high humidity, and wind for a safe flight. In any case, these factors during the inspection may make the flight difficult and even cause interruption.

### 2.2. Data Acquisition

The data acquisition process corresponds to the UAV takeoff and the capture of photographs during the flight. The weather conditions are decisive for the flight. The pilot must ensure the humidity and wind speed are within the manufacturer’s limits for the operation of the UAV. Excessive wind hinders the movement of the aircraft and causes accelerated wear on the battery life, and excessive humidity can damage the device or prevent its operation.

Once the pilot is positioned and the atmospheric conditions are verified, the UAV must be raised and directed towards the structure, alternating the display between the aircraft view and the remote control screen. The latter shows in real time the altitude and flight speed, which are crucial parameters the pilot relies on to maintain a constant speed of displacement at the desired altitude and thus traverse the planar trajectories along each face.

In order to manually capture a sequence of consecutive images separated by a horizontal step, the pilot must maintain a constant horizontal travel speed according to the image displayed on the remote control screen and press the shutter built into the remote control at regular time intervals. The vertical step must be executed after the horizontal sequence in order to move to the next flight line and obtain another horizontal sequence in the opposite direction describing a trajectory, as shown in Figure 3b. The result is a sequence of images, overlapping horizontally with each other and vertically with those of the previous flight line. For the facade, the height parameter of the UAV will be an indicator to raise the UAV one vertical step upward, and on the top face, the UAV must be moved at a constant speed to move one vertical step forward in the flight plane.

Flight planning applications allow the design of automatic flights with the previously determined parameters. However, these should be discarded for two reasons. First, these applications use the GNSS location of the UAV, which is subject to the quality of both the sensor and the perceived satellite signal [53], and this error can lead to accidents when short-range images are needed. Secondly, the quality of the photogrammetric reconstruction improves with the overlap between the images and the completeness of the registration. The calculated parameters of horizontal and vertical pitches should be considered as a minimum for the operator to maintain a constant speed and capture intervals, ideally generating horizontal sequences of images with an overlap greater than the recommendation ideally.

### 2.3. Data Processing

Once the set of photographs for reconstruction is complete, they are processed using the SIFT–SfM–MVS sequence of algorithms. First, in the stage defined as feature extraction, the SIFT algorithm identifies the characteristic points of the scene that can be detected, independent of the scale, illumination, or image orientation [54]. Then, the focal lengths, image sensor size, and device pose serve as input parameters for the SfM algorithm, through iterative processes, to calculate the spatial and georeferenced location of each point. In this step, control points can be included, if available, to improve the georeferencing of the model points; however, with the dimensions associated with short-range reconstructions, the quality of the model will be dependent on the completeness of the scene registration rather than the existence of control points.

Finally, the MVS algorithm densifies the point cloud obtained from the SfM process, improving its visualization and generating a polygonal approximation of the point cloud, and creates a 3D mesh of the scene with high spatial resolution and detailed information of the captured surface [55].

### 2.4. Coordination and Monitoring

This methodology is oriented toward the development of physical progress monitoring assisted by a 4D BIM simulation engine of the construction schedule. Therefore, the development status of the project model in the monitoring of the structure to be reconstructed must be recognized.

Three levels of development are defined for the application of this methodology. The additional work required for its application will depend on the level identified in the project, as described in Table 1.

The assistance of a BIM coordination software is necessary for the implementation of this methodology since tools such as the schedule simulation are available. From the 3D model integrated with the programming (BIM 4D model), a timeline can be created based on a selected date, showing the programmed construction status of the elements of the 3D model, i.e., an as-planned model with the elements under construction and those finished at that date according to the schedule. Thanks to the simulation engine, the timeline can indicate the day coinciding with the data acquisition generated by the UAV, and the as-planned model programmed at that date can be compared with the photogrammetric deliverables from that inspection.

To detect the differences between the planned and the real states of the construction progress, the point cloud delivered by the data processing should be imported into the coordination software to visualize both the as-planned model and the point cloud in the same virtual environment. The comparison can be made by superimposing the point cloud on the 3D model, showing the interaction between the cloud points and the programmed elements in the same spatial location. In the absence of control points and with errors in the GNSS information provided by the device, the SfM reconstruction may incur differences in scale, position, and orientation concerning the original model [18]. To solve this problem, dimensioning tools must first be used to measure distances between the points of the cloud, identify a constructive element in the workspace whose dimensions can be compared with its counterpart in the 3D model, and verify the difference in size between both representations. Then, scaling tools can be used on the point cloud according to the previously obtained dimensions. Finally, the point cloud can be translated and rotated on each of its axes, using easily identifiable reference points and planes, such as corners or vertices between exterior surfaces, to correctly align the 3D model with the point cloud in its three dimensions. Once both representations are superimposed, the scene can be visually explored, and a collision detection tool between specialties can be applied so that the software automatically displays the shared spaces of the point cloud and 3D model.

To identify the constructive state of the elements programmed for the day of the inspection, three states are proposed, as shown in Table 2, to describe the elements aligned with the point cloud.

Surfaces defined by the point cloud that are completely unrelated to the as-planned model can be recognized. These points may correspond to (1) circumstantial elements in the workspace, such as tools and machinery; (2) elements not associated with the schedule used for the 4D coordination; or (3) elements built ahead of the work schedule.

Furthermore, the reconstruction of the 3D mesh obtained from the image processing allows a high-resolution visualization of the worksite. This asset is complementary to the comparison performed in the coordination software and allows for the identification of the condition (1), (2), or (3), in which the groups of points are located in the isolated cloud of the as-planned model. In addition, the construction status of the scheduled elements can be verified in detail, as well as the current situation of those incomplete elements. The digital weight of the 3D mesh is approximately 1/50 of that of the point cloud because the mesh does not work with points but rather with images adapted to the surface formed by the points of the cloud. The mesh represents the reconstructed space through surfaces capable of showing the real colors and textures of the captured space. The high resolution and low digital weight make the 3D map a valuable deliverable of progress monitoring management. Its user friendliness and visualization allow for orthomosaics and views from any perspective of the worksite, which can be integrated into the project communication in meetings or reports to all remote stakeholders and project managers.

## 3. Results

The proposed methodology and workflow were applied to a high-rise construction site for residential use (typical project type in Chile’s current industrial scenario), whose main characteristics are shown in Table 3. The project is located on a hilly topography (hillside), with its back facing a ravine and three levels underground. The building has access to each side from the rear face, and this configuration prevents vehicles from moving along the front of the structure. Some views of the building can be obtained only by means of UAVs. The facade of the structure offers four perpendicular exterior faces, which facilitate the design of trajectories for the flight of the aircraft. This methodology is expected to be extrapolated to projects with more complex shapes, identifying in each case the number of faces and the necessary trajectories for its reconstruction. 

The progress monitoring used on-site was identified at the first level of development; schedule updates were made through visits to the work site supported by a checklist based on the schedule of activities. The construction activities detailed the elements grouped in stages, three stages for the construction of the slabs and five for the walls on each floor of the structure. Each activity, in turn, was divided into a standard sequence of tasks of molding, reinforcement, installations, and concreting for each construction element. Each of the tasks and activities in the schedule had an associated start date and planned duration.

**Figure 4:**
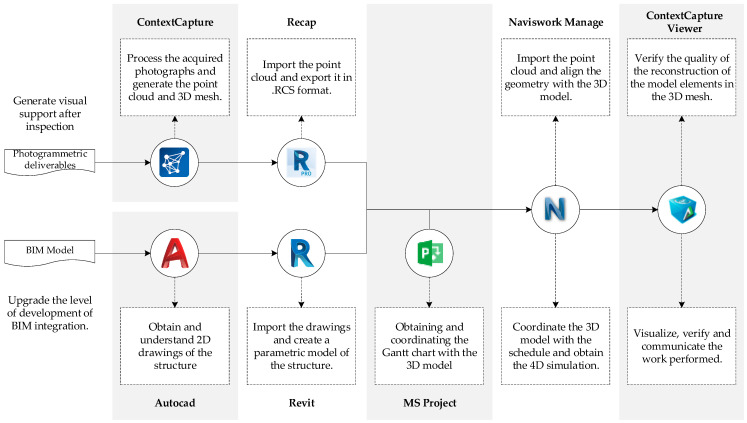
Tools used in the application of the methodology to the case study.

The proposed methodology and workflow present functionalities that can be solved using various options. Different software options are available in the market for the development of BIM models and the application of photogrammetric algorithms for the formation of point clouds and 3D meshes from UAV-acquired images. Figure 4 shows the software used in each stage of the workflow for this case study and their purposes, according to the proposed methodology.

With the absence of the 4D BIM model, a 3D model of the rough work was developed with Autodesk^®^ Revit software from the 2D CAD drawings of the project. The beams, walls, and slabs that structured the building were modeled, respecting the construction sequence and the concordance between elements and the activities of the schedule. For the integration of the schedule, the coordination software Autodesk^®^ Naviswork Manage was used, in which 3D models are imported directly from Revit along with the project’s Gantt chart from Microsoft^®^ Project planning software. In this software, the starting dates of the activities of the schedule were coordinated with the elements of the model, and each task was assigned a color, creating a base file in which the point clouds processed with the records of each inspection would be sequentially imported.

### 3.1. Definition of the Flight Strategy

The surfaces of interest for the reconstruction were identified as the four main faces of the building, belonging to the facade plus the top face, which was located above the working slab. The elements surrounding the project were low-rise constructions, so there was ample safe flying space. Five trajectories could be defined: one for each vertical face (supported by a line of oblique images in front of each vertex) and one for the top face complemented with several lines of oblique images focusing on the working slab.

A representative distance of 15 m was defined for the distance between the UAV and the inspected face, along with 80% horizontal overlap and 65% vertical overlap. Two semi-professional UAVs with similar characteristics were used for the inspections. The camera properties were used to calculate (according to Equations (2) and (3)) the real image size and the horizontal and vertical pitches for each, as shown in Table 4.

**Figure 5:**
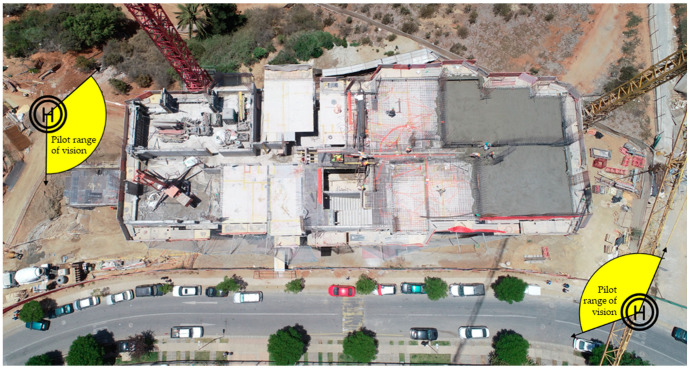
Selected locations for complete inspection of the structure.

Once the vertical and horizontal steps were defined, the most favorable positions for the pilot were identified, considering the height of the working space and the need to maintain visual contact with the UAV. Figure 5 shows the pilot positions and the trajectories designed in front of two consecutive faces of the UAV.

The climatic characteristics of the sector provided variable conditions during the day so that in certain inspections, the humidity or wind limited the UAV flight, preventing the completion of the designed trajectories. The appropriate time for the visit to the worksite and the flight of the aircraft was established at 1 p.m., a decision made based on the following factors:

- For a minimal amount of shadowed areas, flying at hours close to noon was preferable.
- The project location had a humid climate in the early morning hours and experienced an increasing wind speed after noon; this speed was even higher at the height of the working slab, making it difficult for the aircraft to fly.
- The lunch hour for the workers was between 1 p.m. and 2 p.m., leaving the working slab clear of working personnel.

### 3.2. Inspection and Image Acquisition

This methodology is oriented toward the development of physical progress monitoring assisted by a 4D BIM simulation engine of the construction schedule. Therefore, the development status of the project model was determined. The pilot had to adapt to the horizontal and vertical pitches defined in the strategy, depending on the speed of the shutter and the speed of movement of the UAV. To ensure a complete inspection, a manual record was generated using the information on the remote control screens of both devices, maintaining an aircraft displacement speed of 1 m/s and coordinating a shutter release every 3 s, obtaining photographs every 3 m, which satisfied the horizontal pitch margin of both devices. The vertical pitch was monitored using the UAV’s height information on the screens, allowing the continuous acquisition of photographs, following the designed flight trajectories, and generating a set of images representative of the status of the work performed on the day of the inspection.

**Figure 6:**
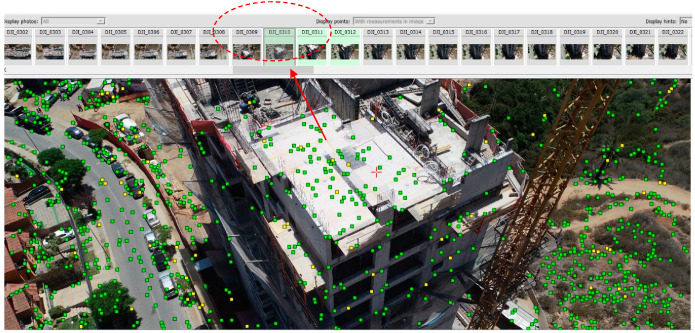
Key points detected in the image set during aerotriangulation.

Six random inspections of the structure were carried out to capture different states of the work progress and compare them with the scheduled progress in the project Gantt chart. For the application of the photogrammetric algorithms and the reconstruction of the 3D scene, Bentley’s Context Capture software was used. This reality modeling program imports the set of photographs and the automatic detection of the camera and poses parameters of each image in the set. Using the aerotriangulation tool, the software applies the key point detection algorithms and identifies the same points in different images of the set. Figure 6 shows the key points detected in a photograph and the number of images in the set in which the same key point is identified. The color of each key point corresponds to the reprojection index of the point, which indicates the accuracy to be reprojected on other images containing it from the aerotriangulation process. In other words, a good reprojection index improves the quality of the aerotriangulation and, thus, of the reconstruction. This color depends on the reprojection error (RMS) of each point:

- High accuracy level (green): RMS < 1 pixel, which ensures good reprojection.
- Medium accuracy level (yellow): 1 pixel < RMS < 3 pixels, and can be used as tie points but with lower quality.
- Low accuracy level (red): RMS > 3 pixels.

**Figure 7:**
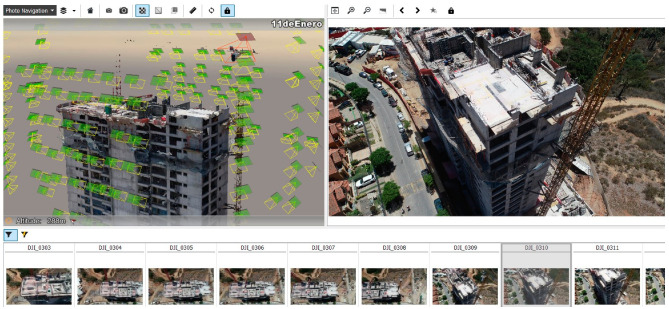
Pose of each photograph rectified by aerotriangulation.

The detection of the key points allows for the triangulation of the positions of each using the pose delivered by the UAV sensors as a starting point of the iteration algorithm. The result of the aerotriangulation is a sparse point cloud with the rectified location and orientation of each photograph obtained by the device (Figure 7).

From the triangulated points and the rectified pose, the 3D mesh was first generated in the native format of the 3MX software to be assessed in the ContextCapture Viewer, and then the point cloud was reconstructed in LAS format to export to the coordination software (Figure 4). Table 5 presents the setting model parameters for the six reconstructions.

### 3.3. 4D Coordination and Identification of Work Performed

Using the base file of the BIM 4D model in the Autodesk^®^ Navis-work software, the point clouds acquired in each inspection day were imported. Since this software has compatibility exclusively with point clouds in the native format of the Recap software of the same firm, the point cloud was imported in LAS format and exported in RCS format to view in the coordination software (Figure 4). The geometric transformation tool was used to translate and rotate each of the reconstructions for correct alignment, verifying that the lateral faces coincided with the outer layer of the point cloud. As mentioned before, the photogrammetric reconstruction may have scale differences with the real dimensions of the structure, which are visible after superimposing both representations. Therefore, the point cloud scaling was adjusted in each import, ensuring that the elements visible in the reconstruction have the same dimensions as those of the 3D model and fixing the point cloud to the lateral faces of the structure. The geometric transformations were applied to each cloud separately, keeping the location of the 3D model intact so as not to affect the visualizations of the other inspections.

To monitor progress over time using the proposed methodology, an isolated record of the work progress in the case study was developed, an isolated record of the work progress in the case study was set as the baseline at day 0, so that each of the following inspections was labeled as the progress of the project from this base date. Thus, starting with the initial inspection as a baseline, the following inspections correspond to days 12, 21, 24, 30, and 41 of scheduled work.

**Figure 8:**
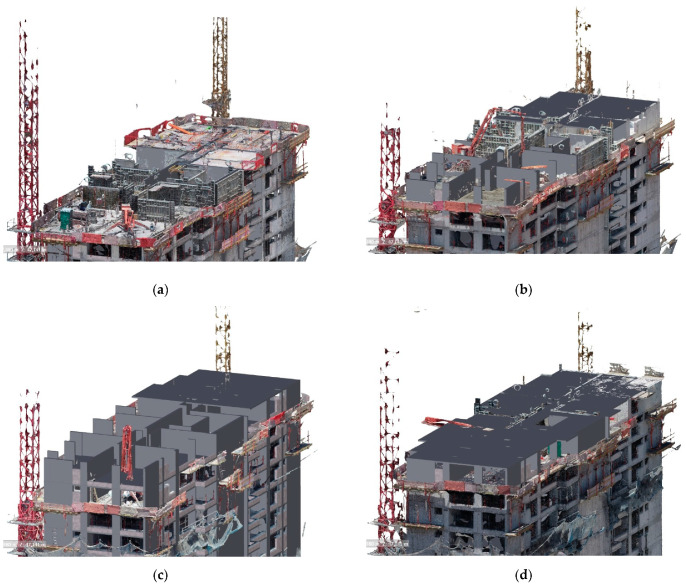

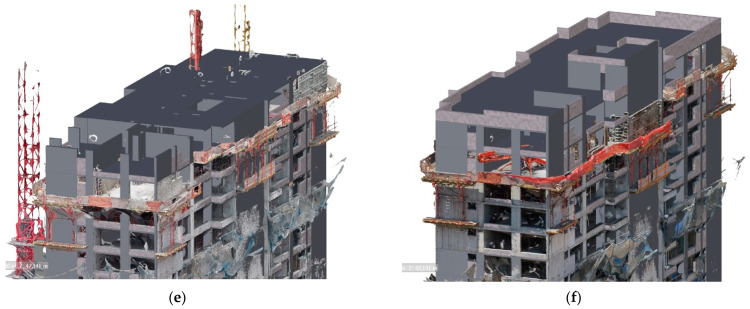
Superposition of point cloud and as-planned model on each inspection day—overlap at (**a**) day 0, (**b**) day 12, (**c**) day, (**d**) day 24, (**e**) day 30, and (**f**) day 41.

Figure 8 shows the superposition of each reconstruction with the 4D model simulation in a base state (**a**) and at 12 (**b**), 21 (**c**), 24 (**d**), 30 (**e**), and 41 (**f**) days of scheduled work. The progress of the schedule was noticeably faster than the actual progress, revealing a delay in the schedule from the second inspection (**b**). From the third inspection (**c**) at 21 days, clearly non-existent elements of the schedule can be seen in the point cloud, indicating a delay of one floor concerning the programmed state of work.

For the identification of the state of the programmed elements, the Clash detection tool (within Autodesk^®^ Navisworks Manage) was used. The interference of the elements belonging to the working slab with the points of the cloud could be detected. The elements that coexist with the cloud and the surrounding points were revealed through a change in the coloring of both representations. The constructive state of the elements would then be determined, as defined in Table 2, as built, unbuilt, or incomplete.

**Figure 9:**
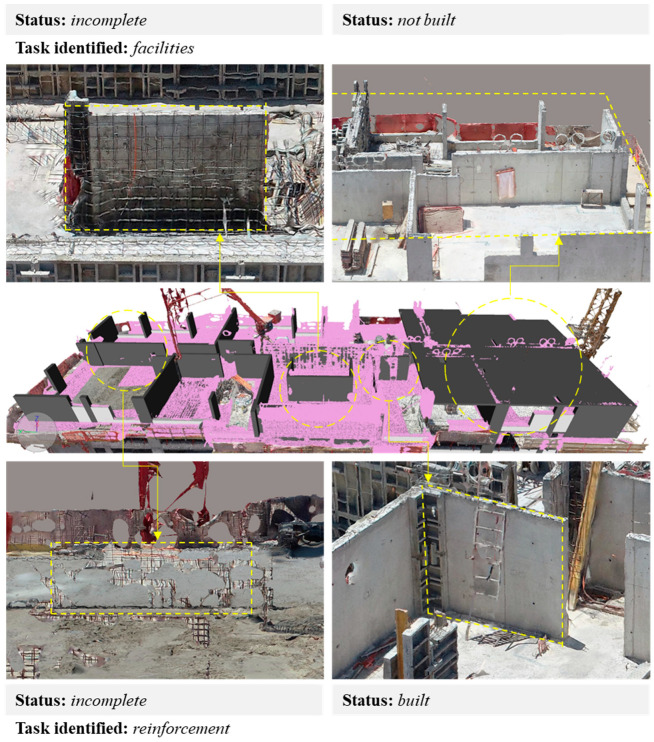
Identification and verification of construction conditions.

The visualization of the point cloud immersed in the coordination software may not have sufficient resolution to verify the real state of the incomplete elements since this is a discrete representation of reality based on points of the captured surface. The representation of thin elements or dimensions close to the GSD of the obtained images was difficult. However, the 3D mesh is a continuous representation of reality, an adaptation of the images obtained on the polygonal surface provided by the point cloud. This representation provides more detailed information of the textures and colors of the real scene and allows better visualization of the elements or separations lower than the resolution of the model. Figure 9 shows that the elements, such as iron and PVC pipes (associated with the tasks of iron posture and installations, respectively), when reconstructed, generated groups of isolated points in the cloud, which were found by Clash detection but had to be visualized in the 3D mesh for a better understanding of their structure.

**Figure 10:**
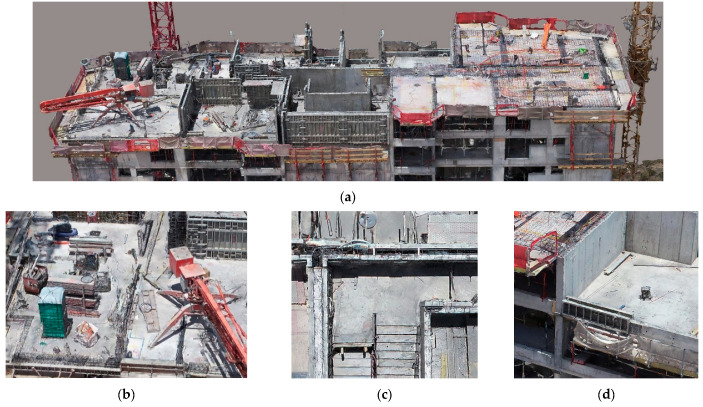
Different aspects observed in the reconstructed model: (**a**) Reality capture of the workspace of the case study, (**b**) stockpiling points and location of tools, (**c**) verification of concreting of elements, (**d**) potentially hazardous areas.

While the methodology integrated the point cloud into the 4D simulation engine to measure the progress of the work, the 3D mesh generated from the image set was a valuable asset that provided detailed information of the workspace scene. The management and visualization of the reality model could produce ortho-rectified images of any surface area of the workspace. Therefore, different attributes associated with the worksite could be visualized, identified, and communicated, allowing progress monitoring, quality control, and safety control remotely, thus improving communication and coordination between project participants. However, the methodology was limited to the technical characteristics of the camera and the potential visibility space provided by the UAV flight strategy. Thus, the methodology should be complemented with other remote monitoring strategies, such as laser scanners, positioning bracelets, and Lidar, among others. Particularly, remote quality control and safety control can be complex to perform with the proposed methodology; therefore, it would be interesting to complement it with video capture strategies in the case of safety management and laser scanners for quality control. Figure 10a shows one of the real models acquired in the inspection, which revealed critical features for workspace management, such as the location of machinery and stockpile points (Figure 10b), the progress of the concreting of the walls (Figure 10c), and sectors without guardrails, potentially dangerous for the personnel in the work area (Figure 10e).

**Figure 11:**
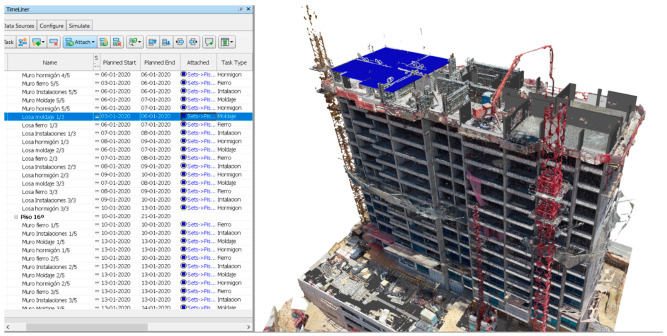
Visualization of the work schedule in the Naviswork Manage software.

With the information collected and the verification of the elements’ constructive states, tracking the scheduled work was possible, assisted by the 4D simulation within the same coordination software. Figure 11 shows a screenshot of the Naviswork Manage software, listing the work schedule and the overlapping of the point cloud and the 4D model from the second inspection. Both the schedule and model shown in blue indicate the task and the element corresponding to a non-existent slab in the point cloud, implying a delayed task that must be rescheduled to maintain an updated work schedule.

The first way to update the schedule was to fix the start date of the task in the original schedule file in the Microsoft Project software. Naviswork scheduling software can include a link to this file for easy synchronization. The second way was to reschedule the start date of the outdated task in the same scheduling software since the concatenation of critical tasks would move them all from a single modification. The main advantage of this second way was to evaluate the impact of a delayed task when it was detected by the point cloud.

## 4. Discussion

The proposed methodology managed to correctly integrate the reconstructed reality models from photogrammetry in the monitoring of the physical progress. The recommendations provided here yielded a manual and orderly acquisition of a photographic record through UAVs for the generation of point clouds and 3D meshes, which provided visual, detailed information of the worksite. The superimposition of the point clouds with the as-planned models in the coordination software interactively compared the statuses of the scheduled work and actual construction. The user could remotely observe, verify, and measure physical progress on the ground. The photogrammetric deliverables can form high-impact views that can be used in meetings, reports, and other project communications to support decision making and improve the quality of the periodic record of project progress.

The photogrammetric techniques used in this methodology showed limitations associated with the quality of the photographic record. Despite the high resolution of the images acquired, the existence of small or thin elements on-site could not be correctly reconstructed in the 3D scene, delivering point clouds and 3D meshes with confusing and incomplete surfaces in these elements’ locations, which resulted in an erroneous representation of the surface. However, the physical characteristics, such as color and shape, in the 3D model defined the types of elements involved and their associated tasks.

Because of the height of the structure of the case study and the geographical conditions of its location, differences in wind speed between the ground level and the height of the worksite were common. This caused complications in the data acquisition from the top face of the structure, restricting the trajectory. However, oblique photographs of the workspace obtained from heights close to the level of the top face contributed to the processing of photogrammetric deliverables of identifying the construction status of the elements of the chronogram. These climatic differences will likely be characteristic of other high-rise buildings to be inspected, depending mainly on the weather of the project site area.

## 5. Conclusions

This research identified problems in the management of physical progress monitoring in construction associated mainly with the generation of visual backups of the current status of the work performed. Construction site photography is a strenuous and subjective task; however, it is very helpful for the measurement of progress, the recording of progress, and the flow of information between the field and project stakeholders. The literature revealed advantages associated with the implementation of three technologies—UAVs, photogrammetry, and 4D BIM models—to complement the traditional approach to physical progress monitoring that allows the generation of useful assets for the registration, measurement, and communication of the work completed on-site.

In this study, the design of a comprehensive methodology was presented with recommendations for the capture and processing of site photographs using UAVs, along with the integration of photogrammetric deliverables to the BIM methodological flow. This methodology establishes a standard for the application of the three technologies studied for the monitoring of the physical progress of a construction project, addressing the shortcomings described in the literature: the breakdown of the different parts of this workflow in isolation and the lack of guidelines for the monitoring of structures by manual flights.

This study proposed the use of UAV devices as an asset that can be integrated into the project management not only to aid in progress monitoring but also for various inspections where access to the worksite is difficult. A designated pilot for a construction project can gain UAV experience and expeditiously generate records, streamlining the flow of information on-site. In this way, construction professionals responsible for analyzing project performance can count on a standard for UAV operation on the job site and the photogrammetric processing of the captured images for project communications and work progress monitoring.

Some limitations of this study are as follows. Since UAVs require a safe flight space, photogrammetric reconstructions using this methodology inside the structures are not possible, so the reality capture is limited to the exterior mantle of the projects to be monitored. The flight of the aircraft is also subject to circumstantial environmental conditions that can hinder a continuous and homogeneous flow of project monitoring.

The accuracy gap between photogrammetry and its reality capture counterpart LIDAR (Laser Imaging Detection and Ranging) laser technology is balanced by the difference in the cost of acquisition and implementation of both technologies, with photogrammetry being a cheaper alternative. However, since both tools use point clouds as deliverables, this study becomes an alternative as well as a complement to LIDAR reality capture systems. Both technologies can be applied for the generation of visual backups of construction sites, providing information through reality models for the monitoring of physical progress, measurement of work performance deviations, quality control, and job site safety remotely, and both can be integrated with the BIM work methodology, generating better coordination and communication between the persons responsible for the project. Additionally, the proposed methodology is limited to the visual space that is possible to capture through a UAV, making it complex to evaluate the progress of project areas that are difficult to access visually. The methodology alone does not allow monitoring the quality and safety of the project, as it is complex to evaluate these variables continuously and with a high level of detail. So, it would be interesting to use other technological and management tools.

Future research should point toward the development of methodologies and computational tools that automate work progress monitoring through the integration of reality capture technologies. The implementation of automation in the different stages of the construction process can quantify its impact on the value chain of the life cycle of a construction project. On the other hand, automating the flight of a UAV allows proper application of flight parameters. However, commercial applications have focused on automating flights on a route over a horizontal plane. Therefore, the challenge arises of how to develop automatic flight plans for surveys over a vertical plane, considering that this dimension (z) is the most complex to represent in a model and where the application of the flight parameters, until today, must be solved only with the expertise of the UAV pilot. The presence of obstacles (as opposed to horizontal flight) is another important aspect that this automation process must consider.

## Figures and Tables

**Table 1:** Modeling work required for the implementation of the proposed methodology.

Level	Development Status	Work Required
1	The project uses only 2D CAD drawings together with the schedule of activities in a Gantt chart format to monitor the physical progress of the work.	Build the 3D model from the 2D CAD drawings of the project using modeling software and associate the start date of each activity in the schedule with the corresponding elements of the model in 4D coordination software.
2	The project has a 3D BIM model representative of the final programmed state of the structure. The monitoring is performed through the usual practices of visiting, recording, and checking through the schedule.	Associate the start dates of the activities in the schedule with the corresponding parametric elements of the 3D BIM model in the coordination software.
3	The project has a 4D BIM model, composed of elements coordinated with the activities of the chronogram through their start and duration dates, allowing visualization of the programmed workspace at any date of the calendar.	Generate the monitoring in the base file of the 4D BIM model.

**Table 2:** Construction status for the elements displayed in the coordination software.

Type of Interaction between the Element and the Point Cloud	Element Status
Element surface coexisting with cloud points.	Built
Volume of the element with cloud points inside or outside it.	Incomplete
Volume of the element without dots inside it.	Not built

**Table 3:** Project characteristics for the case study.

Type of Use	Materiality	Vertical Construction	Height Per Floor [m]	Floor Area Per Floor [m2]	Total Area [m2]	Total Duration [days]
Housing	Reinforced concrete	3 basements 18 floors	2.52	541.6	13,053	420

**Table 4:** Characteristics of the UAV devices used in the case study.

Device	Image Resolution [MP]	Focal Distance [mm]	Sensor Size (h,v) [mm]	Actual Image Size (h,v) [m]	Step (h,v) [m]
Phantom 4 pro	20 (5472 × 3648)	8.8	12.83 × 7.22	21.8 × 12.3	4.3 × 4.3
Parrot Anafi	21 (4068 × 3456)	3.8	5.92 × 5.92	23.4 × 23.4	4.6 × 8.1

**Table 5:** Setting model parameters.

Registration Day	N° 0	N° 12	N° 21	N° 24	N° 30	N° 41
Number of photos uploaded	235	196	200	208	210	190
Number of photos used	210	196	200	208	209	189
Percentage of photos used (%)	89	100	100	100	100	99
Processing time	5 h 42 min	4 h 52 min	4 h 57 min	5 h 10 min	5 h 20 min	4 h 50 min
GSD [mm/px]	9.95	11.96	10.51	10.3	10.9	11.5
Model Scale	1:30	1:36	1:32	1:32	1:32	1:40
Image dimension	5472 × 3078 px	5472 × 3078 px	5472 × 3078 px	5472 × 3078 px	5472 × 3078 px	5472 × 3078 px
Total tie points	60,322	56,597	45,767	50,645	55,455	49,788
Average tie Points per image	1245	1333	1089	1121	1280	1289
Average RMS error	0.47	0.51 px	0.57 px	0.55	0.51	0.5
Minimum RMS error	0.01 px	0.01 px	0.01 px	0.01 px	0.01 px	0.01 px
Maximum RMS error	1.88 px	1.87 px	1.78 px	1.74 px	1.71 px	1.8 px

## Data Availability

Not applicable.
